# Comparative analysis of large language models in the Royal College of Ophthalmologists fellowship exams

**DOI:** 10.1038/s41433-023-02563-3

**Published:** 2023-05-09

**Authors:** Raffaele Raimondi, Nikolaos Tzoumas, Thomas Salisbury, Sandro Di Simplicio, Mario R. Romano, North East Trainee Research in Ophthalmology Network (NETRiON), Tejaswi Bommireddy, Harshika Chawla, Yanmei Chen, Sinéad Connolly, Samy El Omda, Melissa Gough, Lyudmila Kishikova, Thomas McNally, Salman N. Sadiq, Samuel Simpson, Boon Lin Teh, Steven Toh, Vishal Vohra, Mohaimen Al-Zubaidy

**Affiliations:** 1 Sunderland Eye Infirmary, Sunderland, UK; 2 Biosciences Institute, Newcastle University, Newcastle upon Tyne, UK; 3 Department of Ophthalmology, The James Cook University Hospital, Middlesbrough, UK; 4 Newcastle Eye Centre, Royal Victoria Infirmary, Newcastle upon Tyne, UK; 5 Department of Biomedical Science, Humanitas University, Milan, Italy; 6 Cumberland Infirmary, Carlisle, UK

**Keywords:** Education, Outcomes research

## Introduction

Recent years have witnessed an increasing interest in the application of Artificial Intelligence (AI) and deep learning in Ophthalmology [1]. Large language models (LLMs) have become a popular area of research in this field, and have been integrated into publicly available chatbots such as ChatGPT 3.5 and 4.0 (*OpenAI*, CA, US), Google Bard (*Alphabet Inc*., CA, US), and Bing Chat (*Microsoft Corporation*, WA, US) [2–5]. LLMs have been trained on vast amounts of data, enabling them to generate human-like text and answer complex questions. This capability has the potential to revolutionise clinical practice and assessment [2, 6, 7].

We evaluated the performance of LLM-driven AI chatbots on the Fellowship of Royal College of Ophthalmologists (FRCOphth) exams required for autonomous Ophthalmology practice in the UK. We focused on testing the capability of these models in the Part 1 and Part 2 FRCOphth Written exams. These advanced postgraduate exams consist of multiple-choice questions and cover the learning outcomes of the Ophthalmology Specialty Training curriculum in the first two years of training and towards the end of training, respectively.

## Methods

We obtained sample multiple-choice questions from the Royal College of Ophthalmologists website, covering both the Part 1 and Part 2 examinations [8, 9]. We excluded image-based questions, resulting in 48 Part 1 and 43 Part 2 questions, categorised according to their topics. Specialty trainees who had recently passed the exams rated the difficulty of each question on a scale of 1–5, with 1 being “not at all difficult” and 5 being “extremely difficult” (Supplementary Materials). The mean difficulty score was consistent across all respondents.

We tested each LLM-chatbot three times on the sample questions at different timepoints. Additionally, for Part 2 questions, we evaluated ChatGPT-4.0 using various prompting strategies, such as asking the chatbot to answer the question from the perspective of a pharmacist or statistician. When the LLM-chatbot could not answer the question, it was recorded as incorrect. We did not provide additional instruction or training data.

We analysed the association between accuracy and LLM-chatbot using Chi-squared testing and multilevel (mixed effect) logistic regressions. Difficulty and topic were included as fixed effects, and question ID as a random effect. We selected the models with the lowest Akaike information criterion. Part 1 and Part 2 data were analysed separately. All statistical analyses were conducted in R.

## Results

**Fig. 1:**
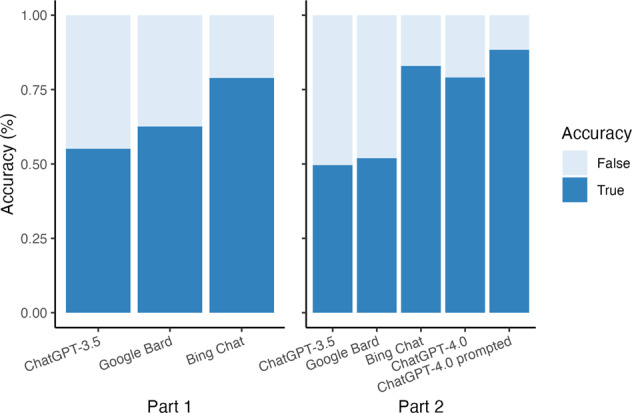
Performance of LLM-chatbots on FRCOphth examinations. The chart displays the average scores obtained by the LLM-chatbots on Part 1 (left) and Part 2 (right) FRCOphth written examinations. The *x*-axis denotes the name of the LLM-chatbots, while the *y*-axis represents the average scores.

The LLM-chatbots achieved overall accuracies of 65.5% and 67.6% for Part 1 and Part 2 questions, respectively (Fig. 1). ChatGPT-3.5, Google Bard, and Bing Chat had respective accuracies of 55.1% and 49.6%, 62.6% and 51.9%, and 78.9% and 82.9% on the sample questions. ChatGPT-4.0 achieved an accuracy of 79.1% on Part 2 questions, which increased to 88.4% with prompting. Significant differences in accuracy were observed between the LLM-chatbots on both question sets (Chi-squared tests *P* < 0.001). Despite a 4% mean difference in accuracy with each iteration, no statistically significant differences in performance were observed for any individual LLM-chatbot.

On multilevel testing, Bing Chat outperformed ChatGPT-3.5 (OR 6.37, 95% CI 3.16–12.83, *P* < 0.001) and Google Bard (OR 3.73, 95% CI 1.88–7.37, *P* < 0.001) in Part 1 questions. No significant associations were found between accuracy and question difficulty or topic. In Part 2 questions, ChatGPT-3.5’s performance was surpassed by both ChatGPT-4.0 and Bing Chat, regardless of whether prompting was used or not (Table 1). LLM accuracy was significantly higher for questions on the “Cornea & External Eye” topic (Table 1). However, we found no other significant associations between LLM-chatbot accuracy and other covariates.

**Table 1:** Comparing the accuracy of responses to FRCOphth Part 2 written questions with different LLM-chatbots.

Covariate	Levels	Inaccurate response	Accurate response	Univariable OR	Multilevel OR
LLM-chatbot	ChatGPT-3.5	65 (50.4)	64 (49.6)	-	-
	Google Bard	62 (48.1)	67 (51.9)	1.10 (0.67–1.79, *p* = 0.71)	1.16 (0.63–2.14, *p* = 0.64)
	Bing Chat	22 (17.1)	107 (82.9)	4.94 (2.82–8.92, *p* < 0.001)***	11.90 (5.54–25.53, *p* < 0.001)***
	ChatGPT-4.0	27 (20.9)	102 (79.1)	3.84 (2.24–6.71, *p* < 0.001)***	8.10 (3.95–16.62, *p* < 0.001)***
	ChatGPT-4.0 prompted	5 (11.6)	38 (88.4)	7.72 (3.10–23.52, *p* < 0.001)***	23.36 (6.51–83.80, *p* < 0.001)***
Difficulty	Mean (SD)	2.8 (0.8)	2.4 (0.8)	0.60 (0.48–0.75, *p* < 0.001)***	0.52 (0.21–1.25, *p* = 0.14)
Topic^a^	Investigations	26 (40.0)	39 (60.0)	-	-
	Trauma	16 (61.5)	10 (38.5)	0.42 (0.16–1.05, *p* = 0.07)	0.11 (0.01–1.92, *p* = 0.13)
	Oculoplastic & Orbit	13 (50.0)	13 (50.0)	0.67 (0.26–1.67, *p* = 0.39)	0.39 (0.03–5.40, *p* = 0.48)
	Glaucoma	24 (61.5)	15 (38.5)	0.42 (0.18–0.93, *p* = 0.035)*	0.13 (0.01–1.41, *p* = 0.09)
	Strabismus	10 (38.5)	16 (61.5)	1.07 (0.42–2.77, *p* = 0.89)	0.68 (0.05–9.31, *p* = 0.77)
	Paediatrics	7 (26.9)	19 (73.1)	1.81 (0.69–5.19, *p* = 0.24)	3.75 (0.23–60.75, *p* = 0.35)
	Retina	23 (29.5)	55 (70.5)	1.59 (0.80–3.21, *p* = 0.19)	1.37 (0.19–10.03, *p* = 0.76)
	Cataract	5 (12.8)	34 (87.2)	4.53 (1.68–14.57, *p* = 0.005)**	2.66 (0.16–45.68, *p* = 0.50)
	Cornea & External Eye	2 (3.8)	50 (96.2)	16.67 (4.60–107.44, *p* < 0.001)***	23.55 (1.42–390.93, *p* = 0.028)*
	Uveitis & Oncology	10 (25.6)	29 (74.4)	1.93 (0.82–4.79, *p* = 0.14)	2.22 (0.23–21.91, *p* = 0.49)
	Neurology	13 (25.0)	39 (75.0)	2.00 (0.91–4.55, *p* = 0.09)	1.59 (0.16–15.53, *p* = 0.69)
	Genetics	6 (46.2)	7 (53.8)	0.78 (0.23–2.66, *p* = 0.68)	1.70 (0.05–61.67, *p* = 0.77)
	Pharmacology	12 (46.2)	14 (53.8)	0.78 (0.31–1.96, *p* = 0.59)	0.89 (0.06–12.26, *p* = 0.93)
	Miscellaneous	14 (26.9)	38 (73.1)	1.81 (0.83–4.05, *p* = 0.14)	1.42 (0.16–12.53, *p* = 0.75)

^a^Effect estimates of question topic on accurate responses, adjusted for LLM-chatbot and difficulty, compared to the reference topic of “Investigations”. Significant differences are indicated by * for *P* < 0.05, ** for *P* < 0.01, and *** for *P* < 0.001. For the performance of individual LLM-chatbots on different topics please see Supplemental Table 2.

*LLM* Large language model, *OR* Odds ratio, *SD* Standard deviation.

## Discussion

This study is the first to demonstrate that publicly available LLM-driven chatbots can consistently provide accurate responses to postgraduate Ophthalmology specialty examinations, achieving an impressive accuracy of up to 82.9% without prompting or instruction tuning. This performance was independent of question topic and difficulty. Notably, most LLMs performed well enough to pass the high standards of these exams, which typically require a score of between 58% and 66% [10, 11]. Previous reports have shown that LLMs can achieve accuracies of up to 67.6% in generalist medical examinations with the use of different training data and instruction prompt tuning [7, 12].

We observed variation in the accuracy of responses between LLM-chatbots (Fig. 1), but each consistently provided similar accuracy with each iteration. Curated prompting strategies enhanced performance. LLMs demonstrated equal proficiency in answering basic science and clinical questions and performed similarly across difficulties and topics, except for Part 2 Cornea/External Eye questions, answered correctly 96% of the time (Table 1). This may reflect the use of different training data by LLMs, as our analyses accounted for question difficulty and characteristics. Limited officially-available questions precluded definitive topic-based comparisons (Supplementary Materials).

Our study has broad implications for the field of Ophthalmology, where large-scale medical AI models are being developed to aid clinical decision-making through free-text explanations, spoken recommendations, or image annotations [2]. LLMs outperformed our specialist examinations, raising questions about the adequacy of traditional assessments in measuring clinical competence. Alternative assessment methods, such as simulations or objective structured clinical examinations, may be needed to better capture the multifaceted skills and knowledge required for clinical practice.

Medical AI technology has great potential, but it also poses limitations and challenges. Clinicians may hold the AI system to a high standard of accuracy, creating barriers to effective human-machine collaboration. Responsibility for the answers generated by these technologies in a clinical setting is unclear; our testing revealed that LLMs could provide incorrect explanations and answers without the ability to recognise their own limitations [6]. Additionally, the use of LLMs for clinical purposes is restricted by inherent biases in data and algorithms used, raising major concerns [2, 6]. Ensuring the explainability of AI systems is a potential solution to this problem, and an interesting research topic. Issues related to validation, computational expenses, data procurement, and accessibility must also be addressed [2].

AI systems will become increasingly integrated into online learning and clinical practice, highlighting the need for ophthalmologists to develop AI literacy. Future research should focus on building open-access LLMs trained specifically with truthful Ophthalmology data to improve accuracy and reliability. Overall, LLMs offer significant opportunities to advance ophthalmic education and care.

## Summary

### What was known before

- Large-scale medical AI models such as Large Language Models (LLMs) are being developed to aid clinical decision-making through free-text explanations, spoken recommendations, or image annotations.
- Previous studies have shown that LLMs can achieve accuracies of up to 67.6% in generalist medical examinations using different training data and instruction prompt tuning.

### What this study adds

- This study is the first to demonstrate that LLMs can consistently provide accurate responses to postgraduate Ophthalmology specialty examinations, achieving an impressive accuracy rate of up to 82.9% without prompting or instruction tuning.
- LLMs outperformed the standards of these specialist examinations, indicating that traditional assessments may not adequately measure clinical competence.
- Issues related to validation, computational expenses, data procurement, and accessibility must be addressed to ensure the safe and effective integration of AI systems into online learning and clinical practice.

### Supplementary information

Supplementary Materials

## Data Availability

Summative data generated or analysed during this study are included in this published article and its supplementary materials. FRCOphth Part 1 and Part 2 Written examination sample questions are freely available online. Difficulty measurements for individual FRCOphth examination questions are available on request.

## References

[1] Jiang X, Xie M, Ma L, Dong L, Li D (2023). International publication trends in the application of artificial intelligence in ophthalmology research: An updated bibliometric analysis. Ann Transl Med.

[2] Moor M, Banerjee O, Abad ZSH, Krumholz HM, Leskovec J, Topol EJ (2023). Foundation models for generalist medical artificial intelligence. Nature.

[3] OpenAI ChatGPT (Mar 13 version) [Large language model] Available at: https://openai.com/blog/chatgpt. [Accessed April 13, 2023].

[4] Bard, an experiment by Google (Mar 21 version). Available at: https://bard.google.com/. [Accessed April 13, 2023].

[5] Microsoft Bing Chat (Feb 7 version). Available at: https://www.bing.com/new. [Accessed April 13, 2023].

[6] Sallam M (2023). ChatGPT utility in healthcare education, research, and practice: Systematic review on the promising perspectives and valid concerns. Healthc (Basel, Switz).

[7] Kung TH, Cheatham M, Medenilla A, Sillos C, De Leon L, Elepaño C (2023). Performance of ChatGPT on USMLE: Potential for AI-assisted medical education using large language models. PLOS Digit Heal.

[8] The Royal College of Ophthalmologists (2016). Part 1 FRCOphth Sample MCQs. Available at: https://www.rcophth.ac.uk/wp-content/uploads/2022/01/Part-1-FRCOphth-Sample-MCQs.pdf. [Accessed April 13, 2023].

[9] The Royal College of Ophthalmologists (2016). Part 2 FRCOphth Written Sample MCQs. Available at: https://www.rcophth.ac.uk/wp-content/uploads/2022/01/Part-2-FRCOphth-Written-Sample-MCQs-20160524.pdf. [Accessed April 13, 2023].

[10] Turner M, Budzynski D, Smith B. Examination Report Part 1 Fellowship of the Royal College of Ophthalmologists (FRCOphth) Examination. (2020). Available at: https://www.rcophth.ac.uk/wp-content/uploads/2021/12/Part-2-Written-Report-July-2021.pdf. [Accessed April 13, 2023].

[11] Budzynski D, Turner M, Smith B. Examination Report and Part 2 Fellowship of the Royal College of Ophthalmologists (FRCOphth) written examination. (2021). Available at: https://www.rcophth.ac.uk/wp-content/uploads/2020/10/Part-1-FRCOphth-Exam-Report_Jan2020.docx. [Accessed April 13, 2023].

[12] Singhal K, Azizi S, Tu T, Mahdavi SS, Wei J, Chung HW, et al. Large Language Models Encode Clinical Knowledge. (2022). Preprint at https://arxiv.org/abs/2212.13138. 10.1038/s41586-023-06291-2 PMC10396962 37438534

