# Supplementary material for: Comparative analysis of large language models in the Royal College of Ophthalmologists fellowship exams
Source: Eye (Lond). 2023 May 9;37(17):3530–3. doi: 10.1038/s41433-023-02563-3 (PMC10686375; doi:10.1038/s41433-023-02563-3)
Supplement: Supplementary file 1 — Supplementary Materials [file 41433_2023_2563_MOESM1_ESM.docx]

**Supplementary Materials**

**Supplemental Table 1.** Performance of LLM-chatbots on questions of varied subject matter and difficulty.

| Exam | Topic | Accuracy, % | Difficulty, median (IQR) |
| --- | --- | --- | --- |
| Part 1 | Pathology | 84.4 | 2.2 (1.8 to 3.2) |
|  | Investigations | 77.8 | 1.2 (1.2 to 1.8) |
|  | Pharmacology & Genetics | 76.2 | 2.5 (1.2 to 2.5) |
|  | Optics | 59.7 | 2.9 (1.8 to 3.3) |
|  | Physiology | 54.4 | 2.5 (2.0 to 2.8) |
|  | Anatomy & Embryology | 52.5 | 2.2 (1.5 to 2.8) |
| Part 2 | Cornea & External Eye | 96.2 | 1.9 (1.6 to 2.3) |
|  | Cataract | 87.2 | 1.4 (1.0 to 1.8) |
|  | Neurology | 75.0 | 2.1 (1.9 to 2.4) |
|  | Uveitis & Oncology | 74.4 | 2.7 (2.3 to 3.9) |
|  | Miscellaneous | 73.1 | 2.4 (2.0 to 2.8) |
|  | Paediatrics | 73.1 | 3.2 (2.6 to 3.8) |
|  | Retina | 70.5 | 2.5 (2.3 to 2.8) |
|  | Strabismus | 61.5 | 2.5 (1.8 to 3.2) |
|  | Investigations | 60.0 | 3.1 (2.8 to 3.2) |
|  | Genetics | 53.8 | 4.6^†^ |
|  | Pharmacology | 53.8 | 3.5 (3.0 to 4.0) |
|  | Oculoplastic & Orbit | 50.0 | 2.7 (1.4 to 3.9) |
|  | Glaucoma | 38.5 | 2.4 (2.1 to 2.6) |
|  | Trauma | 38.5 | 2.5 (1.7 to 3.3) |

^†^Only one FRCOphth Part 2 *Genetics* question was available.

**Supplemental Table 2.** Performance of individual LLM-chatbots on questions of varied subject matter.

| Exam | Topic | LLM-chatbot accuracy, % | | | | | *p*-value^†^ |
| --- | --- | --- | --- | --- | --- | --- | --- |
|  |  | Bard | Bing | GPT-3.5 | GPT-4.0 | GPT-4.0  prompted |  |
| Part 1 | Anatomy and Embryology | 46 | 61 | 52 | - | - | 0.62 |
|  | Investigations | 67 | 100 | 67 | - | - | 0.99 |
|  | Optics | 58 | 88 | 33 | - | - | 0.08 |
|  | Pathology | 87 | 87 | 80 | - | - | 0.48 |
|  | Pharmacology & Genetics | 81 | 100 | 47 | - | - | 0.012 * |
|  | Physiology | 47 | 63 | 53 | - | - | 0.61 |
| Part 2 | Cataract | 78 | 100 | 67 | 100 | 100 | 0.37 |
|  | Cornea & External Eye | 100 | 100 | 83 | 100 | 100 | 0.70 |
|  | Genetics^‡^ | 0 | 100 | 0 | 100 | 100 | 0.08 |
|  | Glaucoma | 33 | 67 | 11 | 33 | 67 | 0.86 |
|  | Investigations | 47 | 73 | 53 | 60 | 80 | 0.45 |
|  | Miscellaneous | 42 | 75 | 92 | 75 | 100 | 0.018 * |
|  | Neurology | 42 | 100 | 50 | 100 | 100 | 0.012 * |
|  | Oculoplastic & Orbit | 100 | 50 | 0 | 50 | 50 | 0.06 |
|  | Paediatrics | 50 | 83 | 50 | 100 | 100 | 0.10 |
|  | Pharmacology | 17 | 83 | 50 | 50 | 100 | 0.21 |
|  | Retina | 61 | 83 | 50 | 83 | 83 | 0.35 |
|  | Strabismus | 33 | 100 | 0 | 100 | 100 | 0.13 |
|  | Trauma | 17 | 33 | 50 | 50 | 50 | 0.19 |
|  | Uveitis & Oncology | 44 | 100 | 44 | 100 | 100 | 0.048 * |

^†^ Results of Kruskal-Wallis rank sum test.

^‡^ Only one FRCOphth Part 2 *Genetics* question was available.
